# Detection and sequence analysis of *Canine morbillivirus* in multiple species of the Mustelidae family

**DOI:** 10.1186/s12917-022-03551-7

**Published:** 2022-12-24

**Authors:** Zsófia Lanszki, József Lanszki, Gábor Endre Tóth, Tamás Cserkész, Gábor Csorba, Tamás Görföl, András István Csathó, Ferenc Jakab, Gábor Kemenesi

**Affiliations:** 1 National Laboratory of Virology, University of Pécs, 7624 Pécs, Hungary; 2 Institute of Biology, Faculty of Sciences, University of Pécs, 7624 Pécs, Hungary; 3 Balaton Limnological Research Institute, 8237 Tihany, Hungary; 4 Hungarian University of Agriculture and Life Sciences, 7400 Kaposvár, Hungary; 5 Department of Zoology, Hungarian Natural History Museum, 1088 Budapest, Hungary; 6 Independent Researcher, 5830 Battonya, Hungary

**Keywords:** Carnivora, Mustelids, NGS, MinION, Third generation sequencing, Protected species, Wildlife disease, Disease ecology, Tailing, Canine distemper virus

## Abstract

**Background:**

*Canine morbillivirus* (canine distemper virus, CDV) is a member of the *Paramyxoviridae* family. Canine distemper is a serious viral disease that affects many mammalian species, including members of the *Mustelidae* family. These animals have an elusive nature, which makes related virological studies extremely challenging. There is a significant knowledge gap about the evolution of their viruses and about the possible effects of these viruses to the population dynamics of the host animals. Spleen and lung tissue samples of 170 road-killed mustelids belonging to six species were collected between 1997 and 2022 throughout Hungary and tested for CDV with real-time RT-PCR.

**Results:**

Three species were positive for viral RNA, 2 out of 64 Steppe polecats (*Mustela eversmanii*), 1 out of 36 European polecats (*Mustela putorius*) and 2 out of 36 stone martens (*Martes foina*); all 18 pine martens (*Martes martes*), 10 least weasels (*Mustela nivalis*) and 6 stoats (*Mustela erminea*) tested negative. The complete CDV genome was sequenced in five samples using pan-genotype CDV-specific, amplicon-based Nanopore sequencing. Based on the phylogenetic analysis, all five viral sequences were grouped to the Europe/South America 1 lineage and the distribution of one sequence among trees indicated recombination of the Hemagglutinin gene. We verified the recombination with SimPlot analysis.

**Conclusions:**

This paper provides the first CDV genome sequences from Steppe polecats and additional complete genomes from European polecats and stone martens. The infected specimens of various species originated from distinct parts of the country over a long time, indicating a wide circulation of CDV among mustelids throughout Hungary. Considering the high virulence of CDV and the presence of the virus in these animals, we highlight the importance of conservation efforts for wild mustelids. In addition, we emphasize the importance of full genomic data acquisition and analysis to better understand the evolution of the virus. Since CDV is prone to recombination, specific genomic segment analyses may provide less representative evolutionary traits than using complete genome sequences.

**Supplementary Information:**

The online version contains supplementary material available at 10.1186/s12917-022-03551-7.

## Background

*Canine morbillivirus* (canine distemper virus, CDV) is a single-stranded, negative-sense RNA virus that belongs to the *Morbillivirus* genus of the *Paramyxoviridae* family [1–3]. The length of the CDV genome is 15,690 nucleotides, and the genome encodes six structural proteins; two glycoproteins: hemagglutinin (H) and fusion (F) proteins, one envelope-associated matrix (M) protein, one nucleocapsid (N) protein, and two transcriptase-associated proteins: phosphoprotein (P) and a large polymerase (L) protein [3, 4]. Several distinct genotypes are known and classified according to different hosts and geographical areas based on nucleotide sequence analysis of the H gene [3, 5–8]. In Hungary, three different CDV genotypes (Europe, Arctic-like and European wildlife lineages) was described so far based on the H gene nucleotide sequences. In addition to dogs, CDV infection was detected in other carnivores, including the red fox and raccoon (*Procyon lotor*), Eurasian otter and ferret (*Mustela furo*) [9–13]. These genotypes have significantly different history, geographic distribution and known host range. Notably, all these CDV is a significant viral pathogen among wild and domesticated animals with a high mortality rate [14, 15]. The virus is primarily transmitted through bodily fluids, e.g., saliva, respiratory droplets, urine, and feces, including transmission due to direct contact [16]. Cross-species transmission occurs frequently, which may lead to conservation problems regarding vulnerable species [17].

Fatal CDV outbreaks are known to occur in wild populations of endangered species. In Africa, CDV caused outbreaks in a diverse range of wild mammals such as the lion (*Panthera leo*), African wild dog (*Lycaon pictus*) and Ethiopian wolf (*Canis simensis*) [14, 18–20]. In Asia, the virus poses a serious threat to the vulnerable Giant panda (*Ailuropoda melanoleuca*) and the endangered Amur tiger (*Panthera tigris altaica*) [21–23]. Additionally, in Europe, CDV infection was also reported in one of the most endangered felid species, the Iberian lynx (*Lynx pardinus*) [24]. In the case of mustelids, CDV infection was previously associated with a high mortality rate approaching 100% [25]. The most remarkable CDV outbreak in black-footed ferret (*Mustela nigripes*) population occurred in Wyoming, Western USA, seriously affecting a captive breeding program and leading to the extirpation of the species from the wild [26, 27]. A recent report from Spain investigated the CDV seroprevalence trends in association to the population size of the Critically Endangered European mink (*Mustela lutreola*). They found that CDV seroprevalence is an indicator for the population trend of these animals, supporting our hypothesis that CDV may be an important wildlife disease [28]. In Europe, CDV has been reported among multiple species to date, including the stone marten (*Martes foina*), pine marten (*Martes martes*), Eurasian badger (*Meles meles*), Eurasian otter (*Lutra lutra*), European mink (*Mustela lutreola*), European polecat (*Mustela putorius*) and the American mink (*Mustela vison*) [13, 29–34].

In Hungary, the Steppe polecat (*Mustela eversmanii*), least weasel (*Mustela nivalis*), stoat (*Mustela erminea*) and pine marten are protected species, the European polecat is periodically considered, and the stone marten is a legally hunted species throughout the year. The stone marten and the European polecat are common, habitat generalists [35, 36]; the least weasel and pine marten are relatively common, whereas the stoat and the Steppe polecat are rare species [37–39]. These mustelids belong to small mammal consumers and omnivorous trophic guilds. Frequent coexistence of up to 5–6 carnivore species and known killings among smaller related species [40] indicate interspecific encounters. These direct contacts may result in cross-infection.

Next generation sequencing (NGS) technologies are increasingly being used to detect and characterize pathogens in wildlife [41–44]. MinION (Oxford Nanopore Technologies, Oxford, UK) has been used in many areas of virology, for instance, metagenomics or sequencing of complete genomes [45–48]. Amplicon-based NGS sequencing of specific pathogens is a method for rapid detection and genomic characterization of target pathogens which may yield high-coverage genomic sequence information [12, 49–52]. With the aid of this technology, we can gain more knowledge about the complete viral genomes, like detection of recombination events.

Detection and investigation of viral diseases are important factors for conserving protected and rare species; however, the elusive nature of several mustelids hampers our understanding of their viruses. Monitoring road-killed animals is a general practice for population genetic studies on rare species [53, 54], but it also gives a good opportunity to get data about pathogens of these animals [13, 55]. Herein we present the results of a post-mortem retrospective surveillance study to detect CDV RNA among road-killed mustelids and perform complete genomic sequencing, phylogenetic and recombination analyses on these virus sequences.

## Results

### PCR screening

*Canine morbillivirus* RNA was detected in three out of the six investigated species: 2 positives out of 64 Steppe polecats, 1 positive out of 36 European polecats and 2 positives out of 36 stone martens. Samples screened from 18 pine martens, 10 least weasels and 6 stoats were negative. The European polecat detected in 2019 and the stone marten in 2020 originated from Western Hungary, both Steppe polecats (collected in 2018 and 2021) originated in Eastern Hungary, and the stone marten (sampled in 2017) was collected in Southern Hungary (Table 1). Two CDV test positive animals (a stone marten and a Steppe polecat) showed signs of bites on their bodies, which indicates combat with another carnivore (Table 1). As the sample collection efforts were not evenly distributed during the study period, CDV prevalence could not be estimated.

**Table 1:** Summary data of CDV-positive mustelids collected in Hungary

Species	stone marten	Steppe polecat	European polecat	stone marten	Steppe polecat
Date of finding	2007	07.12.2018	26.03.2019	01.12.2020	02.05.2021
Tissue	spleen	spleen	lung	spleen	spleen
Age category	juvenile	adult	adult	juvenile	adult
Sex	female	male	female	male	male
Cause of death	road-killed	road-killed	road-killed	road-killed	road-killed
County (settlement)	Somogy	Békés (Battonya)	Vas (Bozsok)	Vas (Felsőjánosfa)	Békés (Nagybánhegyes)
Body condition	poor	good	average	average	good
Other details	-	-	-	bite on the body	bite on the body
RT-PCR Ct value	34.60	38.16	25.61	24.11	46.56
Number of multiplex PCR cycles during sequencing protocol	35	35	27	26	35
Mean sequencing coverage of the targeted region (reads)	11,994.3	3587	17,555.3	2280	102.4
Accession Number	OP209188	OP209186	OP209187	OP209189	OP209185

### Sequencing and phylogenetic analysis

**Fig. 1:**
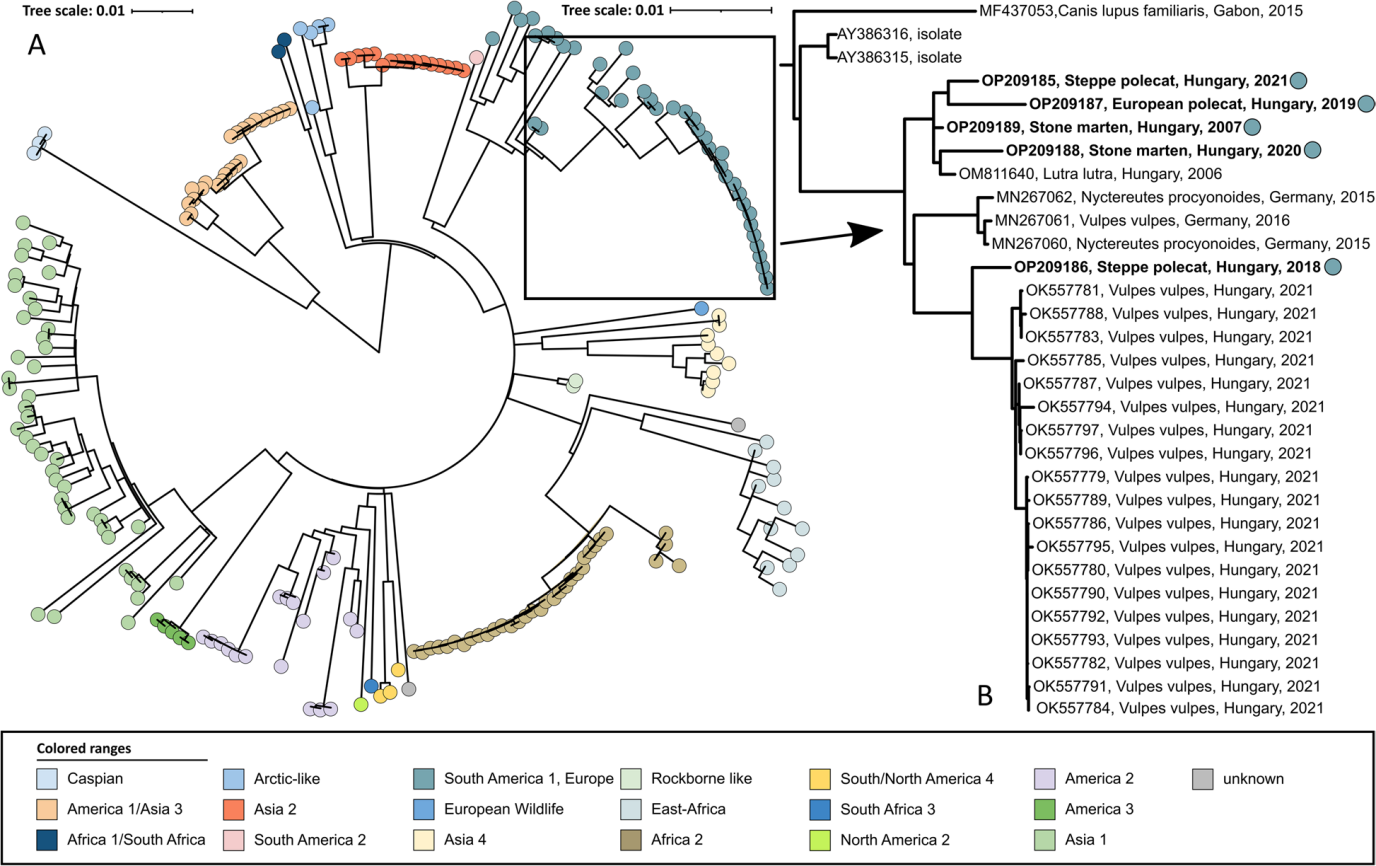
**A** Maximum likelihood phylogenetic tree based on 221 CDV complete genomes. Phocine distemper virus (PDV) (GenBank accession number: KY629928) was used as an outgroup to root the phylogenetic tree. The Europe/South America 1 lineage of interest is highlighted in blue. **B** Expanded portion of Europe/S Am 1 lineage. Dots represent sequences obtained in this study

**Fig. 2:**
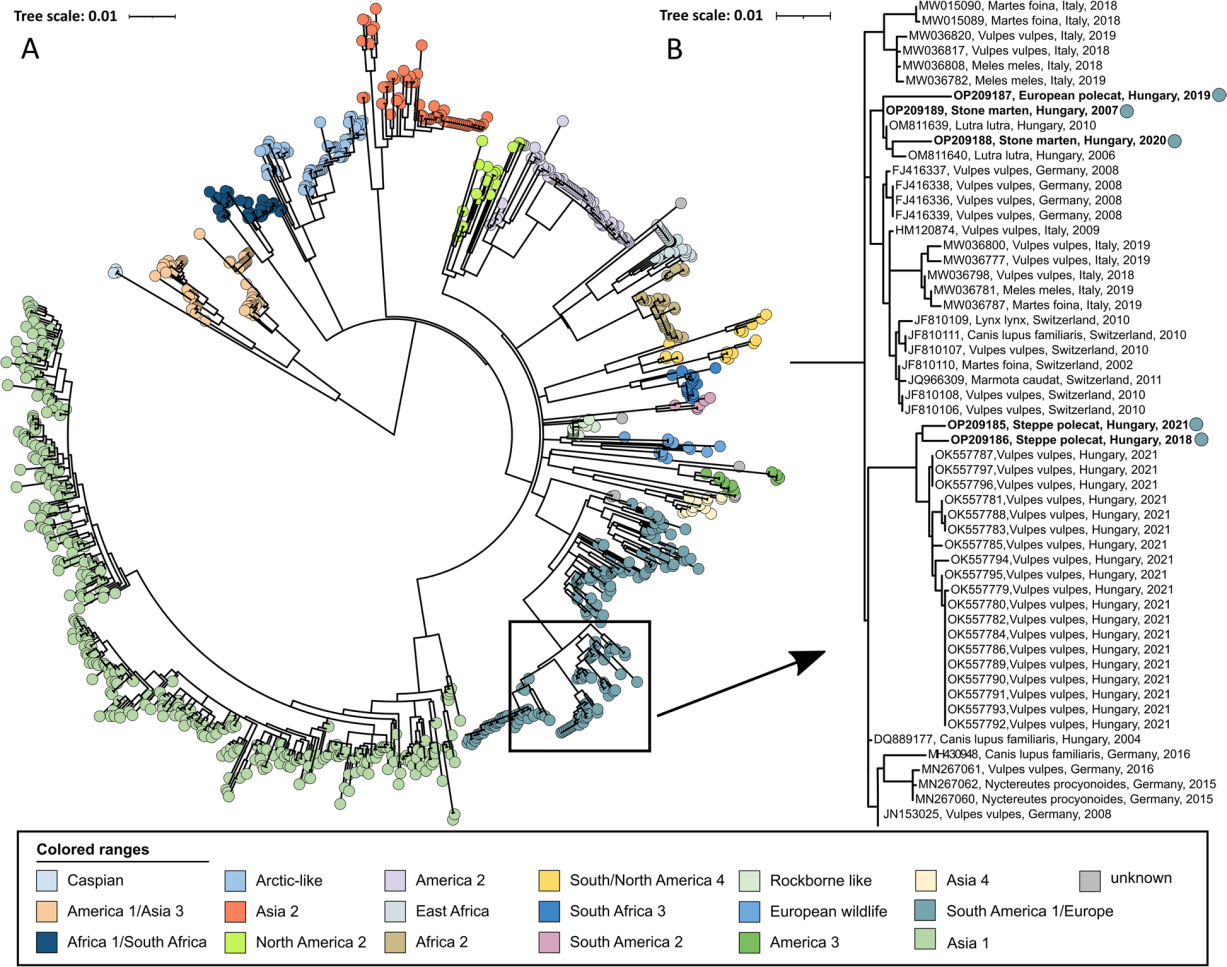
**A** Maximum Likelihood phylogenetic tree based on 969 complete Hemagglutinin (H) nucleotide sequences. Phocine distemper virus (PDV) (GenBank accession number: KY629928) was used as an outgroup to root the phylogenetic tree. The Europe/South America 1 lineage of interest is highlighted in blue. **B** Expanded portion of Europe/S Am 1 lineage. Dots represent sequences obtained in this study

Complete genomes were successfully retrieved from all positive samples. Sequences were deposited in GenBank (accession numbers OP209185-OP209189). Based on the phylogenetic analysis of complete genomes, all these sequences belong to the Europe/South America 1 lineage (Fig. 1). The Hemagglutinin (H) gene sequence-based analysis confirmed this result (Fig. 2).

Sequences are dispersed among two clusters within the Europe/South America 1 lineage, and both clusters are composed of sequences from Hungary. Based on complete genomes (Fig. 1), one cluster contains only mustelid sequences, whereas one Steppe polecat sample was grouped with red fox (*Vulpes vulpes*) samples in a separate clade. Based on the H gene phylogenetic tree, both Steppe polecat samples (OP209186) grouped with red fox samples on a distant clade (Fig. 2). The secondary analysis with RAxML plugin for Geneious supported the primary phylogenetic patter of the sequences, all main lineages and the novel sequences were positioned similarly (Supplementary material; Supplementary Figs. 2 and 3).

**Fig. 3:**
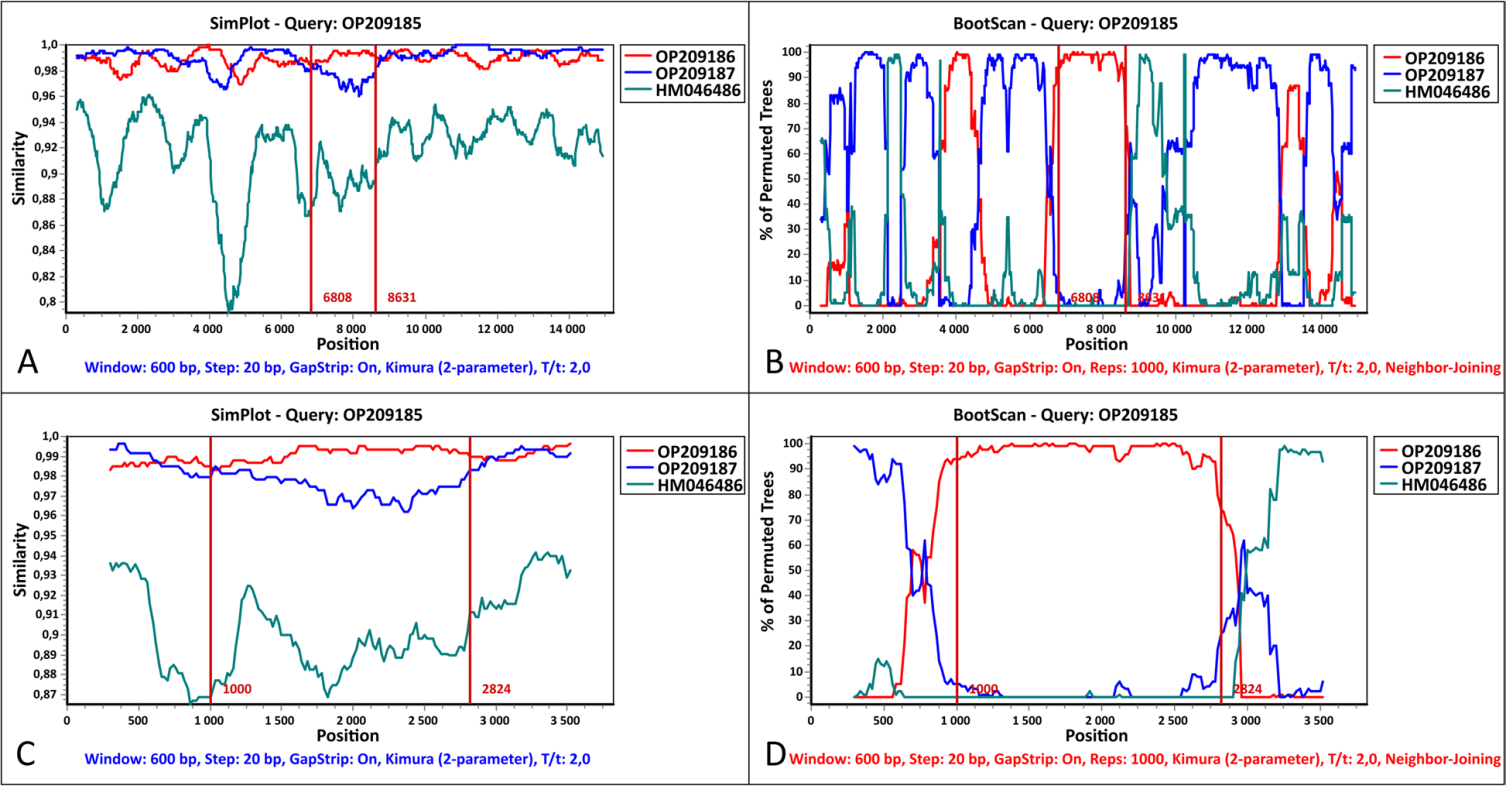
Recombination analysis of the canine distemper virus (OP209185): (**A**) Similarity Plot analysis of the complete genome sequences of OP209185 (Steppe polecat, Hungary, 2021) and its putative parents OP209186 (Steppe polecat, Hungary, 2018) and OP209187 (European polecat, Hungary, 2019). The OP209185 was used as the query. (**B**) Boot Scan analysis of OP209185 and its parent sequences. (**C**) Similarity Plot analysis of the H gene sequences of OP209185 and its putative parents. (**D**) Boot Scan analysis of OP209185 and its parent sequences in the H gene. A CDV isolate, HM046486 (Caspian lineage), was used as an outgroup in all analyses. The red vertical line represents the H gene segment region. The y-axis indicates the percentage of identity with a window size of 600 bp and a step size of 20 bp. The comparison was performed using 50% consensus sequences with 1000 bootstrap replicates

The distinct clustering pattern of OP209185 from a Steppe polecat on the phylogenetic trees (Fig. 1 and 2) indicates a recombination event in association with the Hemagglutinin genomic region. The SimPlot analysis confirmed the recombination of the Hemagglutinin gene with a closely related, Europe/South America 1 lineage strain. Also, it confirmed multiple additional recombination points in the genome (Fig. 3). The secondary analysis with DualBrothers plugin in Geneious also detected multiple recombination points throughout the genome with the same crossing-points (Supplementary material; Supplementary Fig. 4).

## Discussion

We present the circulation of CDV throughout the country over several years, supporting the endemic nature of this virus among mustelids. An important finding of the current study is the detection of CDV in wild-living Steppe polecats. It is a rare and protected mammal species of our region and by using retrospective virus surveillance methods (i.e. without disturbance and invasive sampling of the animals), we were able to indicate the role of these animals in CDV transmission. Steppe polecat was already a suspected host for CDV [56]; however, due to its rareness and elusive nature, only a few molecular biological investigations have been performed on this species without presenting viral genomic data [30]. In the current study we present the first two complete CDV genomes from the Steppe polecat, enriching the diversity of available CDV genomes. By revealing the presence of a recombinant CDV strain in these animals we demonstrated the importance of generating complete genomic data. This approach may ultimately lead to better understanding CDV evolution, since partial genome fragments are not suitable to understand the impact of recombination events in CDV evolution or the role of coding regions other than H. Furthermore, the presence of CDV was confirmed in two additional species in this study. The European polecat is also at risk of infection by CDV; for instance, the virus was detected with RT-PCR (qPCR) from the Asturias region of Atlantic Spain in 2021 [57]. The stone marten is a well-known host of CDV, and in recent decades, many cases have been detected in nearby countries including such as Austria, the Czech Republic, Germany, Switzerland and Italy [29–31, 58–61].

According to our findings and previous literature data, CDV is present in 4 out of the 8 species of the *Mustelidae* family in our region [9, 10, 13, 40]. Considering the relevance of these animals in conservation biology, vaccination in wildlife rescue centers may be an important tool in the conservation of rare and protected mustelids [62]. For instance, the black-footed ferret, which population has almost been extinct due to CDV infection, is a close relative of the Steppe polecat. The vaccination of black-footed ferret × Steppe polecat hybrids was reported as surrogates for endangered black-footed ferrets [63, 64]. In Europe, CDV was detected in Spain in four carnivore species collected in 2020–2021, including the Eurasian badger, pine marten, European polecat and the red fox [57]. In the Czech Republic, CDV was detected between 2012–2020 in the red fox, stone marten, raccoon, pine marten and the European badger [59]. Similar outbreaks were observed among red foxes across Europe due to this strain [12, 60, 65–67]. Europe/South America 1 lineage was also detected in many other species such as Iberian wolves (*Canis lupus signatus*), an Asian marmot (*Marmota caudata*) kept in a zoo, a stone marten, pine marten, Eurasian lynx (*Lynx lynx*), Iberian lynx and a domestic dog [24, 31, 42, 68–70].

For effective transmission of CDV, close contact among infected and susceptible animals is necessary. Bites on two positive animals (stone marten and Steppe polecat) were observed as a direct indication of contact with other carnivores. Aggressive intra- and interspecific behavior are relatively common in the mustelid species, and competition for territory [71], food, or mating partner can effectively facilitate the spread of the disease. Nonetheless, according to published literature, skin contact, feces or urine are less important means of transmission [22, 51, 52]. However, the primary method of transmission in CDV infection is theorized to be via the respiratory tract droplets [72, 73], which may have relevance under fighting conditions. More studies and observational data are necessary to better understand the natural transmission and circulation patterns of CDV.

Based on literature data, the Europe/South America 1 lineage of CDV, which circulates among mustelids throughout Hungary, is also present in surrounding countries [31, 59, 60, 66, 67]. Similar to most of the CDV surveillance studies, H-gene phylogeny was a useful tool for lineage categorization. However as a major limitation, H-gene based analysis is not adequate to reveal genome-scale recombination patterns and understand fine-scale evolutionary patterns. Based on literature data these viruses are prone to recombine in several genomic regions, most frequently in the H gene [74, 75]. We support this with our observation and presentation of multiple recombination points in our recombinant CDV strain. More complete genomic sequence data in the future can reveal a more accurate evolutionary scenario for our sequence. In addition the dispersive pattern among these two phylogenetic clades, composed by different CDV strains from other animal species raises the possibility for cross-species transmission events. This was already known from literature data as an important feature of CDV transmission [76, 77].

A limitation of our study is the lack of autopsy or histology data to better understand the pathogenicity of the CDV infection in these animals. Further studies are needed to discuss the pathogenic nature of these different CDV strains. However, our study highlighted the importance of genome-scale monitoring of CDV evolution, which may serve as a first step to understand genomic evolution in relation to pathogenesis. In addition to these, our study demonstrated that road-killed carcasses are a valuable source of CDV surveillance in wildlife species.

## Conclusion

Understanding the long-term presence of CDV in free-living mammals is of great importance, especially among mustelids, which are particularly sensitive to CDV. As we demonstrated in our study, retrospective sample surveillance coupled with complete genomic sequencing are useful tools to understand the host range of CDV and describe a more detailed evolutionary picture of the virus. Amplicon-based NGS methods are ideal tools to gain complete genomes even from organ samples stored over a long time and most importantly from samples with low viral titers.

## Methods

### Sample collection

Road-killed mustelids (*n* = 170) were collected in Hungary between 1997 and 2022 by the staff of National Park Directorates and volunteers and stored at -20 °C until processing. Tissue samples from spleen and lung via general dissection procedures were collected from the Steppe polecat (*n* = 64), European polecat (*n* = 36), stone marten (*n* = 36), pine marten (*n* = 18), least weasel (*n* = 10) and stoat (*n *= 6) [see Supplementary material; Table 1, Fig. 1]. The post-mortem examination was carried out by the Carnivore Ecology Research Group at the Kaposvár Campus of the Hungarian University of Agriculture and Life Sciences [78] and by the Hungarian Natural History Museum, Budapest [36, 37, 79]. We scored the body condition based on fat deposit over flanks between 1 (poor), 2 (average) and 3 (good) [80]. Tissue samples were stored at -20 °C in the Kaposvár Campus. A few months before nucleic acid extraction, they were deposited in the National Laboratory of Virology at -80 °C.

Research and sample collection permits were issued by the relevant authorities to the Kaposvár Campus (SO-04Z/TO/392–2/2019) and to the Hungarian Natural History Museum (14/6156/7/2011, OKTF-KP/6903–21/2015, PE-KTF/736–6/2017, PE-KTFO/329–16/2019, PE-KTFO/1568–18/2020, PE-KTFO/1403–3/2022).

### Nucleic acid extraction and PCR reactions

For most animals, nucleic acids were extracted from the spleen, but lung was substituted when spleen was not available. Tissue samples were homogenized in 500 μl phosphate buffered saline (PBS), using a TissueLyser LT device (Qiagen, Hilden, Germany) at maximum speed for three minutes, supplemented with two glass beads per sample to facilitate tissue disruption. The total RNA was extracted using the Monarch Total RNA Miniprep Kit (NEB, USA) in full adherence to the manufacturer’s recommended guidance. The samples were screened with a CDV-specific real-time RT-PCR method [3] using OneStep RT-PCR Kit (Qiagen, Germany). RNA was added to each tube and the cycling was adjusted to one cycle of 50 °C for 30 min for the reverse transcription of RNA to cDNA, followed by one cycle at 95 °C for 15 min. The cDNA was amplified by PCR for 50 cycles, each cycle consisting of denaturation at 94 °C for20 sec, annealing at 46 °C for 30 s, extension at 72 °C for 30 s and final extension at 72 °C for 10 min. RT-PCRs were performed immediately following RNA extraction without freeze-thawing the nucleic acid to avoid RNA degradation.

### Nanopore sequencing and data analysis

The complete genome sequencing was performed with MinION Nanopore sequencing technology (Oxford Nanopore Technologies, UK). We used a previously published universal amplicon-based sequencing method designed for CDV [12, 13]. The detailed protocol and the primers are available at our laboratory protocols.io page [81]. In brief, the CDV RNA positive nucleic acids were used for cDNA preparation with Superscript IV Reverse Transcriptase (Invitrogen, USA) using random hexamers. Two sets of primers were used to generate overlapping genome fragments that differ in the length of amplicons (1000 bp, 2000 bp). These multiplex PCRs were conducted directly from the cDNA with the usage of Q5 Hot Start HF Polymerase (New England Biolabs, USA). For the cleanup step, we used AMPure XP beads (Beckman Coulter, USA), and the PCR products were end-prepped with NEBNext Ultra II End Repair/dA-Tailing Module (New England Biolabs, USA). Barcodes from EXP-NBD196 (Nanopore Technologies, UK) were ligated to generate amplicons with NEBNext Ultra II Ligation Module (NEB, USA). The sequencing runs were performed on a R9.4.1. (FLO-MIN106D) flow cell with the AMX-F motor protein from SQK-LSK110 kit (Nanopore Technologies, UK). Sequencing raw data was processed by regular methods for Oxford Nanopore sequencing. Base-calling and demultiplexing of the raw data was performed with Guppy software (version 6.0.1.) using the super accurate base-calling model and default parameters with the “barcode_both_end” option. The generated reads were further processed, as 50 bases pairs were trimmed from both ends and the dataset was filtered to eliminate the short and chimeric sequence reads. Following the previously mentioned processes, all generated reads from a sample were mapped to the MN267060 reference sequence using Geneious Prime (version 1.6.0.). The preconsensus sequences were polished with Medaka (version 2022.1.1) to generate final consensus sequences.

### Phylogenetic and recombinant analysis

Prior to the phylogenetic reconstruction, sequences of interest were retrieved from GenBank (NCBI, Bethesda, USA) and aligned with our obtained sequences in MUSCLE alignment webserver. Two datasets were used for phylogenetic tree analysis comprising 221 complete genomes and 969 complete hemagglutinin gene sequences, respectively. Subsequently, the Maximum Likelihood phylogenetic tree was constructed under the General Time Reversible Model, Gamma Distributed with Invariant Sites (GTR + G + I) substitution model with best model selection in MEGA X (MEGA, Pennsylvania, USA) [82]. The clustering of the sequences was verified with and additional method, using the RAxML (Randomized Axelerated Maximum Likelihood) plugin for Geneious Prime® 2022.2.2 [83]. The resultant tree was edited in iTOL (iTOL, Heidelberg, Germany) [84]. Phocine distemper virus (PDV) was used as an outgroup for all phylogenies.

The potential recombinant CDV genomes were tested through recombination analysis using similarity plot and bootscan analyses in SimPlot software package (version 3.5.1.) [85]. The recombination analysis was modeled with Kimura 2-parameter distance model using a window size of 600 bp and step size of 20 bp in the case of complete genomes and H gene sequences. To support our observation, we used a secondary recombination analysis method with the DualBrothers plugin in Geneious Prime® 2022.2.2 [86].

## Supplementary Information

**Additional file 1.**

## Data Availability

Sequence data are deposited under GenBank accession numbers OP209185, OP209186, OP209187, OP209188 and OP209189. All data analyzed during this study are included in the results section.

## Abbreviations

CDV: Canine distemper virus
PDV: Phocine distemper virus
PBS: Phosphate buffered saline
