# Supplementary material for: Detection and sequence analysis of Canine morbillivirus in multiple species of the Mustelidae family
Source: BMC Vet Res. 2022 Dec 24;18:450. doi: 10.1186/s12917-022-03551-7 (PMC9789673; doi:10.1186/s12917-022-03551-7)
Supplement: Supplementary file 1 — Additional file 1. [file 12917_2022_3551_MOESM1_ESM.pdf]

Supplementary Table 1 Sample numbers of Mustelids tested for *Canine morbillivirus* virus in Hungary. The years 1998, 1999, 2001, 2013, 2014 and 2015 are not included in the table, there was no sample from these years.

	Steppe polecat	European polecat	Stone marten	Pine marten	Least weasel	Stoat
1997	-	-	1	-	-	-
//						
2000	-	-	-	-	1	-
//						
2002	-	-	-	-	2	-
2003	-	-	1	-	-	-
2004	-	-	4	-	-	-
2005	-	1	-	-	1	-
2006	-	-	4	-	1	1
2007	-	1	8/1	-	1	-
2008	2	1	1	-	2	-
2009	-	1	2	-	-	-
2010	1	-	-	-	-	-
//						
2012	-	-	1	-	-	-
2014	-	-	-	2	-	-
2016	2	-	-	-	-	1
2017	3	3	-	-	1	-
2018	10/1	5	2	2	-	1
2019	8	11/1	1	2	1	1
2020	12	4	5/1	5	-	-
2021	18/1	7	5	5	-	2
2022	8	2	1	2	-	-
**Total/Positive**	**64/2**	**36/1**	**36/2**	**18/0**	**10/0**	**6/0**

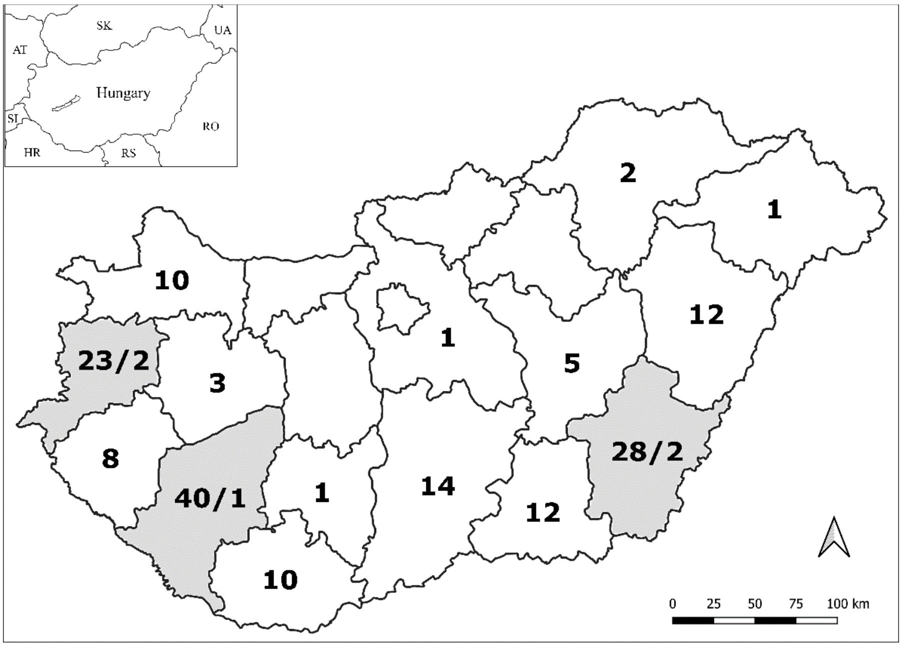

Supplementary Fig. 1 Regional breakdown of the sample numbers, in Hungary (Total/Positive).

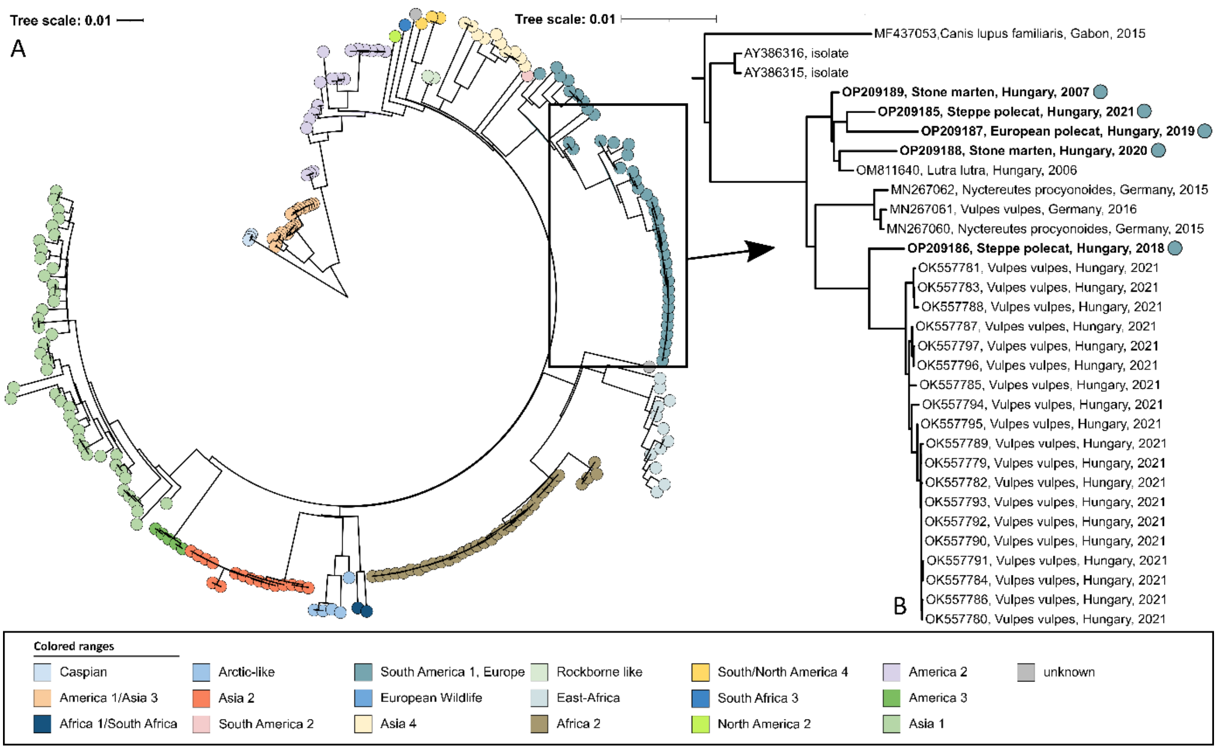

Supplementary Fig. 2 (A) Maximum likelihood phylogenetic tree based on 221 CDV complete genomes, using the **Randomized Axelerated Maximum Likelihood (RAxML)** tool. Phocine distemper virus (PDV) (GenBank accession number: KY629928) was used as an outgroup to root the phylogenetic tree. The Europe/South America 1 lineage of interest is highlighted in blue. (B) Expanded portion of Europe/S Am 1 lineage. Dots represent sequences obtained in this study.

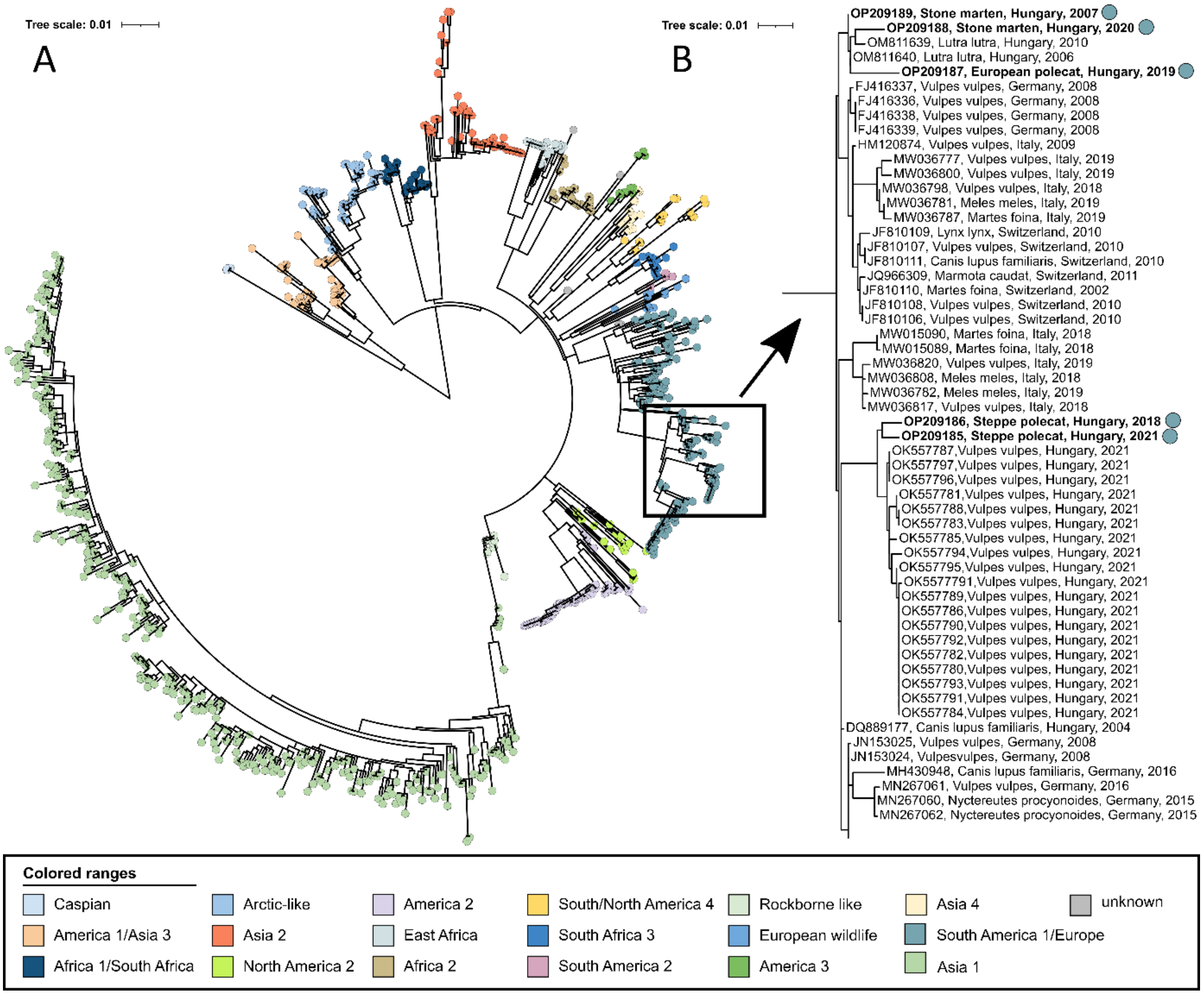

Supplementary Fig. 3 (A) Maximum Likelihood phylogenetic tree based on 969 complete Hemagglutinin (H) nucleotide sequences, using the **Randomized Axelerated Maximum Likelihood (RAxML)** tool. Phocine distemper virus (PDV) (GenBank accession number: KY629928) was used as an outgroup to root the phylogenetic tree. The Europe/South America 1 lineage of interest is highlighted in blue. (B) Expanded portion of Europe/S Am 1 lineage. Dots represent sequences obtained in this study.

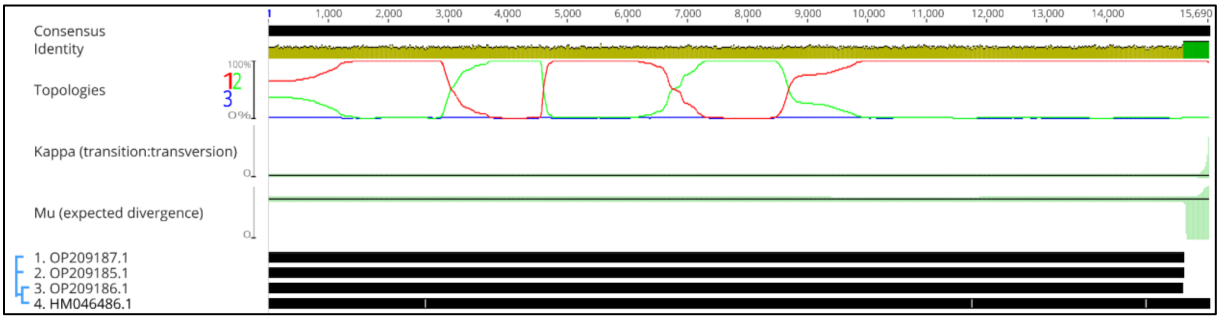

Supplementary Fig. 4 Recombination analysis of the canine distemper virus (OP209185) with Geneious Prime® 2022.2.2. program, DualBrothers plugin. A CDV isolate, HM046486 (Caspian lineage), was used as an outgroup in all analyses. The y-axis indicates the percentage of identity with a window length 200 bp and a step size of 10 bp.
